# Recovered Energy from Salinity Gradients Utilizing Various Poly(Acrylic Acid)-Based Hydrogels

**DOI:** 10.3390/polym13040645

**Published:** 2021-02-22

**Authors:** Tri Quang Bui, Vinh Duy Cao, Wei Wang, Anna-Lena Kjøniksen

**Affiliations:** 1 Faculty of Engineering, Østfold University College, P.O. Box 700, 1757 Halden, Norway; quang.t.bui@hiof.no (T.Q.B.); vinh.d.cao@hiof.no (V.D.C.); 2 Department of Chemistry & Center for Pharmacy, University of Bergen, P.O. Box 7803, 5020 Bergen, Norway; wei.wang@uib.no

**Keywords:** hydrogel, salinity gradient, polyelectrolyte, recovered energy

## Abstract

Hydrogels can be utilized to extract energy from salinity gradients when river water mixes with seawater. Saline-sensitive hydrogels exhibit a reversible swelling/shrinking process when they are, alternately, exposed to fresh and saline water. We present a comparison of several poly(acrylic acid)-based hydrogels, including poly(acrylic acid) (PAA), poly(acrylic acid-*co*-vinylsulfonic acid) (PAA/PVSA), and poly(4-styrenessulfonic acid-*co*-maleic acid) interpenetrated in a poly(acrylic acid) network (PAA/PSSA-MA). The hydrogels were synthesized by free radical polymerization, copolymerization, and by semi-IPN (interpenetrating polymer network). The hydrogels were placed in a piston-like system to measure the recovered energy. Semi-IPN hydrogels exhibit a much higher recovered energy compared to the copolymer and PAA hydrogel. The recovered energy of 60 g swollen gel was up to 4 J for the PAA/PSSA-MA hydrogel. The obtained energy per gram dried gel was up to 13.3 J/g. The swelling volume of the hydrogels was maintained for 30 cycles without decline in recovered energy.

## 1. Introduction

Developing renewable energy sources [1] and reducing greenhouse gas emissions [2,3] are essential to avoid global warming. Clean and affordable alternative energy sources [4,5,6,7] have started to replace fossil energy in many respects of our daily life. One of the clean energy sources is salinity gradient energy, which is generated when solutions with different salinities are mixed [8,9]. The salinity gradient energy when river water flows into the ocean has been estimated as equal to a 200 m high waterfall [9]. Globally, this corresponds to more than 1 TW, enough to cover 1% of the energy demand on the planet [10,11,12].

Salinity gradient energy is the Gibbs free energy of mixing (∆G_mix_), which is lost as entropy increase when mixing two miscible liquids [4]. Salinity gradient power generation technologies can convert ∆G_mix_ to electrical power, and includes pressure-retarded osmosis (PRO) [10,13,14,15,16,17], reverse electrodialysis (RED) [18,19,20,21,22,23], and capacitive mixing (CapMix) [24,25,26,27,28]. PRO employs a semipermeable membrane, which separates the flows of the low and high salinity solutions. The difference in chemical potential and osmotic pressure generates a water flow across the membrane, which feeds a hydro-turbine to generate electricity. RED uses cation and anion exchange membranes, which selectively allow the transport of counter-ions and retains co-ions of the separate solutions of different salinity. The difference in ion concentration at the membranes results in an ion flux, which is then converted to electricity by redox reactions at the electrodes [4]. CapMix principally works as an electrochemical double-layer capacitor. A pair of active carbon porous electrodes is charged in the high salinity solution under an external electric potential. The electrodes are then discharged by an external load resistor in the low salinity solution [4,25]. CapMix is a newer technology compared to PRO and RED, and the power capacity is therefore currently lower, whereas loss of efficiency induced by membrane fouling and the high cost of the PRO and RED technologies has restricted their use.

The alternative of using hydrogels to convert salinity gradient energy into mechanical energy has been introduced in recent years [29,30,31,32]. High and low salinity solutions alternatingly flow through a hydrogel, which is placed in a modified syringe operating in a piston-type process. In freshwater, the electrostatic repulsions between charged groups cause the hydrogel to swell. When the swollen hydrogels are exposed to a high salinity solution, the salt causes a salting-out effect as well as a screening of charges on the polymer chains, resulting in a shrinkage of the hydrogels. Hydrogels have the advantage of being cheap, easier to keep, and are more controllable than membrane-based technologies. The first studied hydrogels for this application was poly(acrylic acid) [29,30]; later, poly(allylamine hydrochloride) [31] and zwitterionic polysulfobetaine [32] hydrogels have been examined. It has been found that the energy efficiency decreases with increasing particle size [29,32] and goes through a maximum as the crosslinking density [30,31,32] or external load [29,30,31] is increased. At high external loads, the recovered energy decreases for hydrogels with many charged groups [30] or low crosslinking densities [30,31].

Polymer hydrogels can swell and retain large amounts of water up to hundreds of times their dry weight [33,34,35,36,37,38,39]. Dynamic mechanical properties during the swelling and shrinking process has been reported to be the main factors that enable hydrogels to lift an external heavy weight [29,31]. Increasing the crosslinking concentration leads to enhancement of the mechanical properties [40,41,42], but reduces the swelling ratio of the hydrogels [40,43]. Drawbacks of chemical hydrogels, in terms of mechanical properties, have been circumvented by adding nanoparticles to the polymer network or by utilizing double- or semi-interpenetrating networks [44,45,46,47,48,49]. The salinity responsiveness also makes hydrogels interesting for other applications such as desalination of fresh water from seawater [50,51], and separating various types of oil and aqueous solutions over a wide range of salinities [52].

Gel swelling is highly dependent on the balance of electrostatic forces and the entropic elastic force in a hydrogel, and therefore affect the amount of energy that can be stored by the hydrogel. Due to their high charge density, polyelectrolytes are especially suitable for applications where a high swelling ratio is desired. In this work, we explore the application of hydrogels for generating energy from mixing saltwater and freshwater, and present a system to examine the energy recovery ability of the hydrogels. While previous studies have focused on one type of polymer, three different types of hydrogels are compared in this study. Such a comparison is important, since it can be difficult to compare the energy efficiency of hydrogels from different studies, as the recovered energy is also strongly dependent on the experimental setup [29]. A series of hydrogels based on poly(acrylic acid) (PAA), containing sulfonic groups as copolymers or in semi-interpenetrating polymer networks (semi-IPN) were synthesized by free radical polymerization. The carboxylic acid groups on poly(acrylic acid) have K_a_ = 5.6 × 10^−5^; while sulfonic acid with K_a_ = 2 × 10^−1^ dissociates completely in water in a wide pH range [53,54]. Introducing sulfonic acid groups in PAA hydrogels is therefore expected to significantly improve the swelling of the hydrogels. Interestingly, the hydrogels exhibited the ability to lift 5 kg weights for long times in repeated cycles utilizing a small amount of dried material.

## 2. Materials and Methods

### 2.1. Materials

Acrylic acid (AA), *N’,N’*-methylenebis(acrylamide) (MBA), ammonium persulfate (APS), *N,N,N’,N’*-tetramethylethylenediamine (TEMED) and poly(4-styrene sulfonic acid-*co*-maleic acid) sodium salt (PSSA-MA) with an average molecular weight of 20,000 g/mol were purchased from Sigma-Aldrich (Merck KGaA, Darmstadt, Germany) and used as received. Vinylsulfonic acid sodium salt (VSA, Sigma-Aldrich, Merck KGaA, Darmstadt, Germany) was used as received as a 25% wt. aqueous solution in water containing 100 ppm of the inhibitor 4-methoxyphenol (MEHQ).

### 2.2. Synthesis of PAA Gels, IPN PAA/PVSA Gels and Semi-IPN PAA/PSSA-MA Gels

**Scheme 1:**
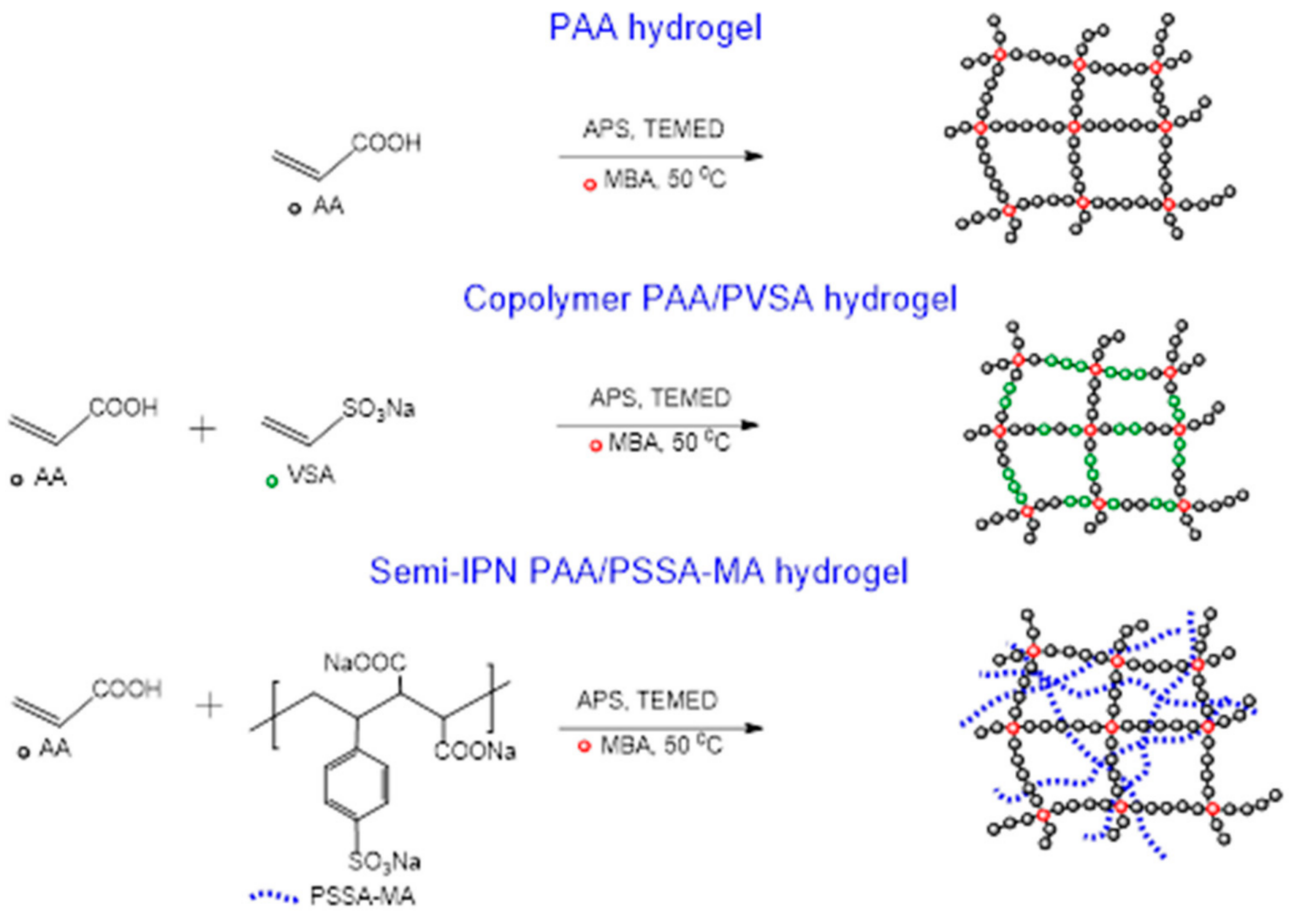
The preparation of the hydrogels.

A series of PAA-based hydrogels were prepared by free radical polymerization in de-ionized water. Three types of hydrogels were prepared: PAA gels, PAA/PVSA gels, and PAA/PSSA-MA gels, with molar crosslinking concentrations of 1.5%, 3.0%, 4.0% (see Scheme 1). The synthetic process was the same for all gel samples to ensure that the results are comparable. The concentrations of monomers are listed in Table 1. Since the strong electrostatic interactions of sulfonic acid groups give rise to a retardation in growth of polymer chains during propagation, the gelation time of PAA/PSSA-MA and PAA/PVSA are longer than for PAA.

Monomer solutions were prepared by mixing 2.5 g monomer in 20 mL distilled water. The TEMED, APS, and MBA was consecutively added to the monomer solutions under stirring. The mixture was purged with nitrogen gas for 10–20 min to remove oxygen, after which the gelation was carried out in an oven at 50 °C. After gelation, the hydrogel was cut into small pieces and immersed in a large quantity of de-ionized water to remove unreacted monomers and catalysts. The dialysis water was changed daily for 15–20 days in order to reach equilibrium swelling for the hydrogels. The dialysis was carried out at room temperature (approximately 22 °C).

### 2.3. Salinity Gradient Setup and Operation

**Figure 1:**
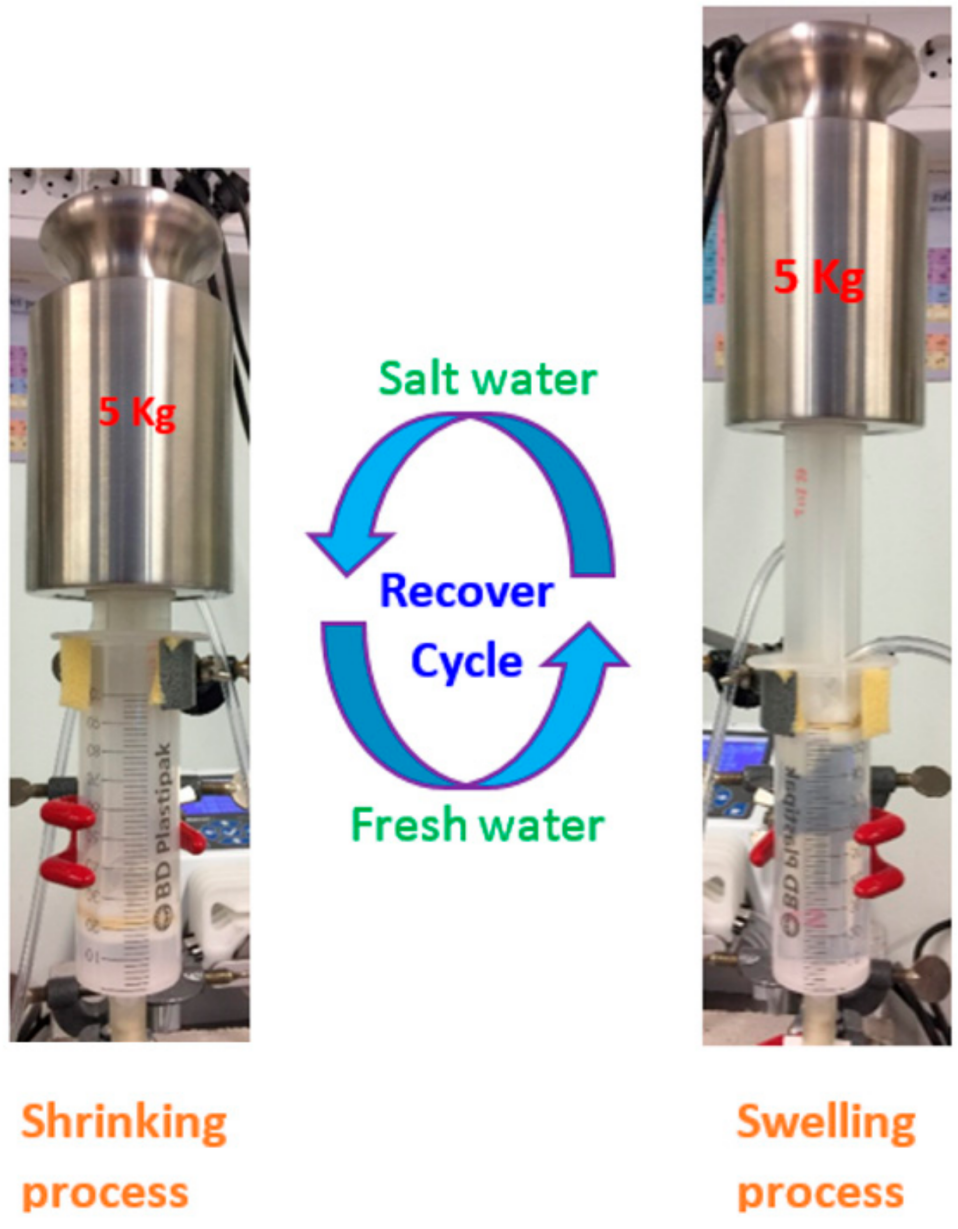
Illustration of a cycle of the energy recovery process.

A modified syringe and plunger were utilized to examine the recovered salinity gradient energy, as described in our previous work [31]. The diameter and volume of the syringe are 3.5 cm and 100 mL, respectively. The syringe was placed vertically, to pump the saline solutions from the bottom to the top. Four holes of approximately 1 cm in diameter were drilled through the bottom of the plunger to facilitate the flow of water. The holes were covered by small cloths, to prevent the hydrogel from being pressed through the holes when applying a heavy weight. Figure 1 illustrates the cycling of the energy recovery process for the system. A saline solution (35 g/L NaCl, ionic strength 0.599 M) and de-ionized water were alternately pumped through the hydrogel, from the bottom of the syringe.

Hydrogels were fully swollen in de-ionized water for at least 15–20 days before initiating the measurements. The fully swollen gel (60 g) was placed in the syringe and a load weight of 5 kg was applied on the top of the plunger. The high salinity solution was pumped at a rate of 3 mL/min from the bottom of the syringe through the hydrogel.

The excess flow-out solution was pumped out at the holes of the plunger. When the plunger had moved downward to the minimum volume due to the shrinking of the hydrogel, the system was exchanged to pumping de-ionized water at a rate of 5 mL/min. The flow rate was controlled by peristatic pumps (Reglo ICC, Ismatec, Cole-Parmer GmbH, Ismatec, Wertheim, Germany). The hydrogel swells, lifting the 5 kg weight to the maximum level. Each cycle, including one shrinking and one swelling, lasted for one hour. The cycles were repeated 6 times in a day. The experiments were carried out for 5 consecutive days for a total of 30 cycles.

### 2.4. Swelling Ratio

The swelling ratio (*Q*) is expressed as:

*Q* = *m_s_*/*m_d_* (1)

where *m_s_* is the mass of the fully swollen hydrogel (approximately 60 g), and *m_d_* is the mass of the dried hydrogel. At least three repetitions were carried out by using at least two batches of hydrogels.

### 2.5. Oscillatory Shear Rheology

The rheological properties of the hydrogels were carried out on a rheometer (Anton Paar MRC 302, Graz, Austria) using a parallel plate geometry of 50 mm in diameter. The swollen hydrogels were synthesized in a chamber with the same geometry as the test chamber in the rheometer. We followed the same synthetic procedure as for the swelling experiments. After gelation, the gel sheets were transferred to a water bath for swelling in de-ionized water for 14 h. The diameter of the resulting hydrogel sheets was 70–80 mm after 14 h swelling, depending on the type of hydrogel. The hydrogel sheets were carefully transferred to the rheometer. The storage modulus (*G’*) and loss modulus (*G’’*) were measured in an oscillatory frequency sweep experiment. The measurement was conducted in the linear viscoelastic regime with a strain amplitude of 0.01%, and frequencies ranging of 1 to 100 rad/s.

### 2.6. Structure Characterization

The hydrogel structures were examined by Fourier transform infrared spectrometer (FTIR, Perkin-Elmer, Waltham, MA, USA, spectrum BX) in the wavelength of 4000 to 500 cm^−1^, with steps of 4 cm^−1^, and 32 scans.

### 2.7. Recovered Energy

The recovered energy (*E*) is defined as the total potential energy of the external load W_1_, which is lifted at a height of h_1_ by the swelling of the hydrogel. The work done with regards to the upward movement of the center of gravity of the hydrogel is *W_2_*. The recovered energy was calculated as:

(2) $$E=W_{1}\;+\;W_{2}=\;m_{1}g_{0}h_{1}+\;m_{2}g_{0}h_{2}$$

where *g*_0_ (9.81 m/s^2^) is the gravitational constant, *h*_1_ is the displacement of the plunger calculated based on the expanding volume and the diameter of the syringe (*D*), *h*_2_ = *h*_1_/2 is the displacement of the center of gravity of the hydrogel, *m*_1_ is the external load, and *m*_2_ is the average weight of the swollen and de-swollen hydrogels calculated according to volumes, assuming the density of the hydrogel is 1000 kg/m^3^.

### 2.8. Energy Efficiency

The energy efficiency (*η_E_*) was calculated as the percent ratio of the recovered energy to the energy consumed by the system, which is the change in the free energy due to a complete mixing of the high and low salinity solutions.

(3) $$\eta_{{}_{E}}=\frac{E}{X^{in}-X^{out}}\times 100$$

where *X^in^* is the total energy provided to the system, and *X^out^* is the total energy leaving the system, which is equal to 0. *X^in^* which was calculated as:

(4) $$X^{in}=RT\sum^{}\left(V_{HC}c_{i,HC}\ln\frac{\alpha_{i,HC}}{\alpha_{i,M}}+V_{LC}c_{i,LC}\ln\frac{\alpha_{i,LC}}{\alpha_{i,M}}\right)$$

where *R* (8.314 mol^−1^ K^−1^) is the gas constant, *T* (298 K) the temperature, *V* the volume of the solution, *c_i_* the molar concentration of the ionic species *i* in the solution, and *α_i_* the activity of the ionic species *i* in the solution. The subscript *M* indicates the mixed solution; while subscripts *LC* and *HC* indicate the low and high concentrations of NaCl, respectively. The recovered energy and energy efficiency were computed from the average values of the last 6 cycles for each measurement. It should be noted that energy supplied to the system by the pump that facilitates the flow of the high and low salinity solutions is not included in these calculations.

## 3. Results and Discussion

### 3.1. Swelling Ratio of Hydrogels

Copolymer hydrogels PAA/PVSA and semi-IPN PAA/PSSA-MA hydrogels exhibit a large increase of swelling ratios compared to the PAA hydrogel. At pH = 7, the carboxylic groups in PAA partially dissociate, whereas the sulfonic acid dissociates completely. The charged sulfonic acid groups generate strong repulsive forces in the hydrogel, causing a higher swelling capacity [55,56,57]. On the other hand, high amounts of intermolecular hydrogen bonds in the PAA network restricts the swelling of the PAA hydrogel. In the presence of PVSA or PSSA-MA, it is more difficult for the PAA-chains to arrange close to each other for formation of hydrogen bonds. The swelling ratios of the PAA/PSSA-MA semi-IPN network is higher than for the PAA/PVSA copolymer hydrogel at the same crosslinker concentration due to the flexible mobility of the non-crosslinked interpenetrating hydrophilic polymer in PSSA-MA. The interpenetrating network (Scheme 1) provides enhanced mechanical strength to the hydrogel (enabling it to lift heavy weights), without restricting its swelling ability in the way that a crosslinked network does.

### 3.2. Rheological Properties of the Hydrogels

**Figure 2:**
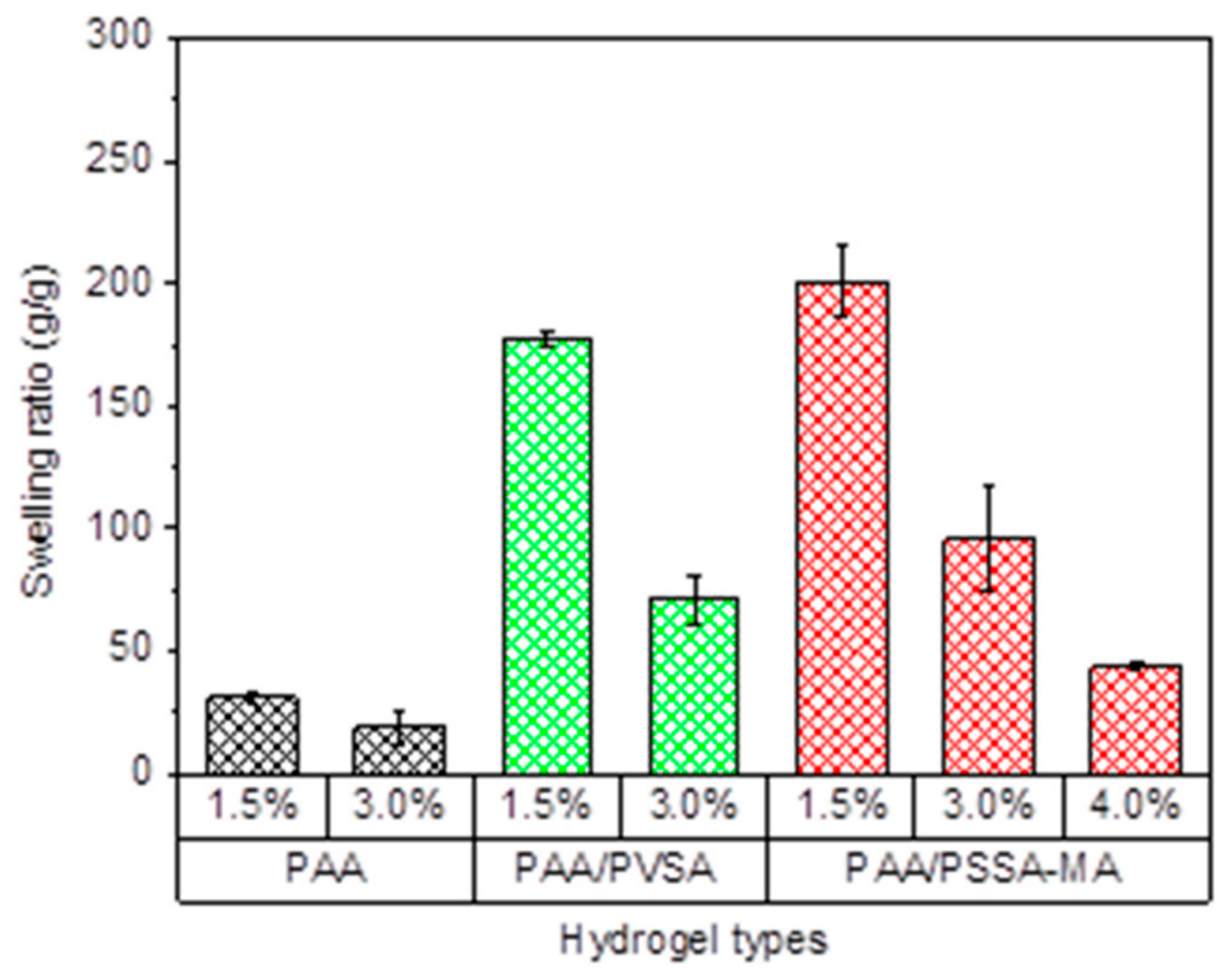
Swelling ratio of the different hydrogel groups: poly(acrylic acid) (PAA), copolymer (acrylic acid-*co*-vinyl sulfonate sodium) (PAA/PVSA), semi-IPN of poly(4-styrene sulfonic acid-*co*-maleic acid) sodium salt in PAA network (PAA/PSSA-MA). The percentages indicate the crosslinking density.

**Figure 3:**
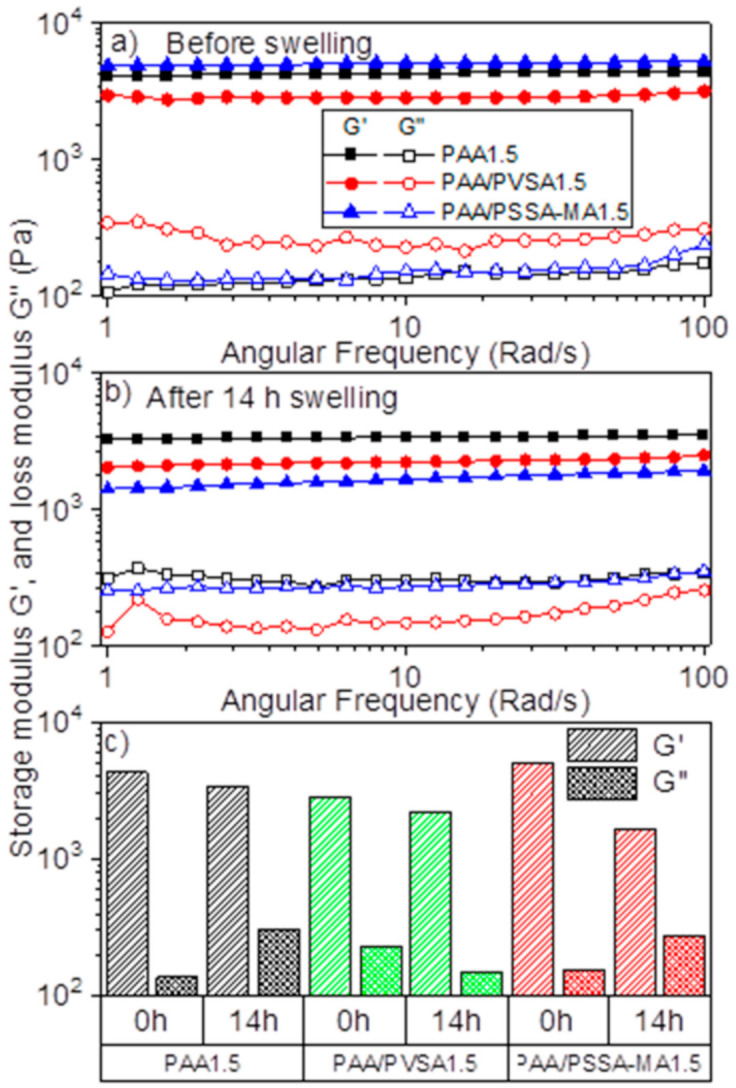
Storage modulus (G’) (solid symbol) and loss modulus (G’’) (open symbol) of hydrogels of PAA1.5, PAA/PVSA1.5, and PAA/PSSA-MA1.5: (**a**) hydrogel before swelling; (**b**) hydrogel after swelling for 14 h; (**c**) at an oscillatory frequency of 10 rad/s before (0 h) and after (14 h) swelling.

In order to lift a heavy load for generating high amounts of recovered energy, the hydrogel network needs to have a strong mechanical structure, which is also capable of swelling highly in water. However, while increasing the crosslinker concentration enhances the mechanical properties of the network structure, it also decreases swelling ratio [31]. Including strongly charged functional groups (e.g., sulfonic acid) in the low-swelling PAA network is expected to increase the swelling ratio (as was observed in Figure 2) while maintaining the structural strength. This is confirmed by the rheological measurements of the swollen hydrogels (Figure 3a,b). The rheological properties of the initial hydrogels after preparation and without further swelling are shown in Figure 3a. The storage modulus (*G’*) of the semi-IPN hydrogel (PAA/PSSA-MA1.5) is higher than for PAA1.5 and PAA/PVSA1.5. This can be attributed to an enhancement of hydrogen bonds due to the high amounts of SO_3_^−^ and COO^–^ groups in PSSA-MA [57,58]. In addition, the hydrogel is reinforced by entanglements of PSSA-MA within the PAA network.

**Figure 4:**
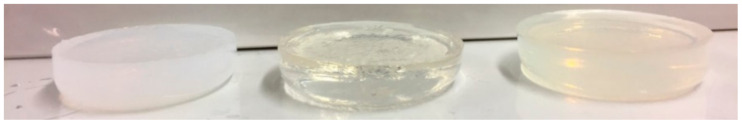
Image of the hydrogels: PAA1.5 (left); PAA/PVSA1.5 (middle); PAA/PSSA-MA1.5 (right).

Interestingly, PAA/PVSA1.5 has a lower storage modulus than PAA1.5, suggesting that copolymerizing with PVSA weakens the PAA network. This is in agreement with Nesic et al. [57], where the storage modulus was found to decrease when 2-acrylamido-2-methylpropane sulfonic acid was introduced to a poly(methacrylic acid) hydrogel. The presence of PVSA sodium salt in a random copolymer may cause a change from intermolecular to intramolecular hydrogen bonds. It is worth noting that intermolecular hydrogen bonds are formed between carboxylic acid groups in the PAA and PSSA-MA backbones in the semi-IPN PAA/PSSA-MA hydrogel, thereby enhancing the mechanical properties of the PAA/PSSA-MA hydrogel compared to the PAA hydrogel (Figure 3c). This effect is not observed for the PAA/PVSA hydrogels, which exhibits reduced mechanical properties compared to pure PAA hydrogels. The differences in hydrogen bonds is corroborated by Figure 4, where the PAA1.5 hydrogel is much more turbid than the PAA/PSVA1.5 hydrogel, while the PAA/PSSA-MA1.5 has a turbidity between the two other hydrogels. The turbidity is caused by compact micro-domains and inhomogeneities in the hydrogel. As shown in connection with the discussion of Figure 7, these microdomains in the PAA1.5 gel are caused by hydrogen bonds.

After swelling in de-ionized water for 14 h, the storage modulus (*G’*) of the PAA/PSSA-MA1.5 hydrogel decreased much more than for PAA1.5 and PAA/PVSA1.5 (Figure 3b). This is partly due to the higher swelling of PAA/PSSA-MA1.5 (Figure 2). Enhanced swelling reduces effective polymer concentration within the hydrogels, thereby making them weaker. In addition, PAA/PSSA-MA1.5 is a semi-interpenetrating network. Accordingly, some of the polymer chains are not crosslinked, but just entangled within the crosslinked network (Scheme 1). While this makes the network stronger in the de-swollen state, a high degree of swelling might reduce the effectiveness of the entanglements, thereby significantly decreasing the mechanical properties of the hydrogel.

### 3.3. FTIR Spectrum

**Figure 5:**
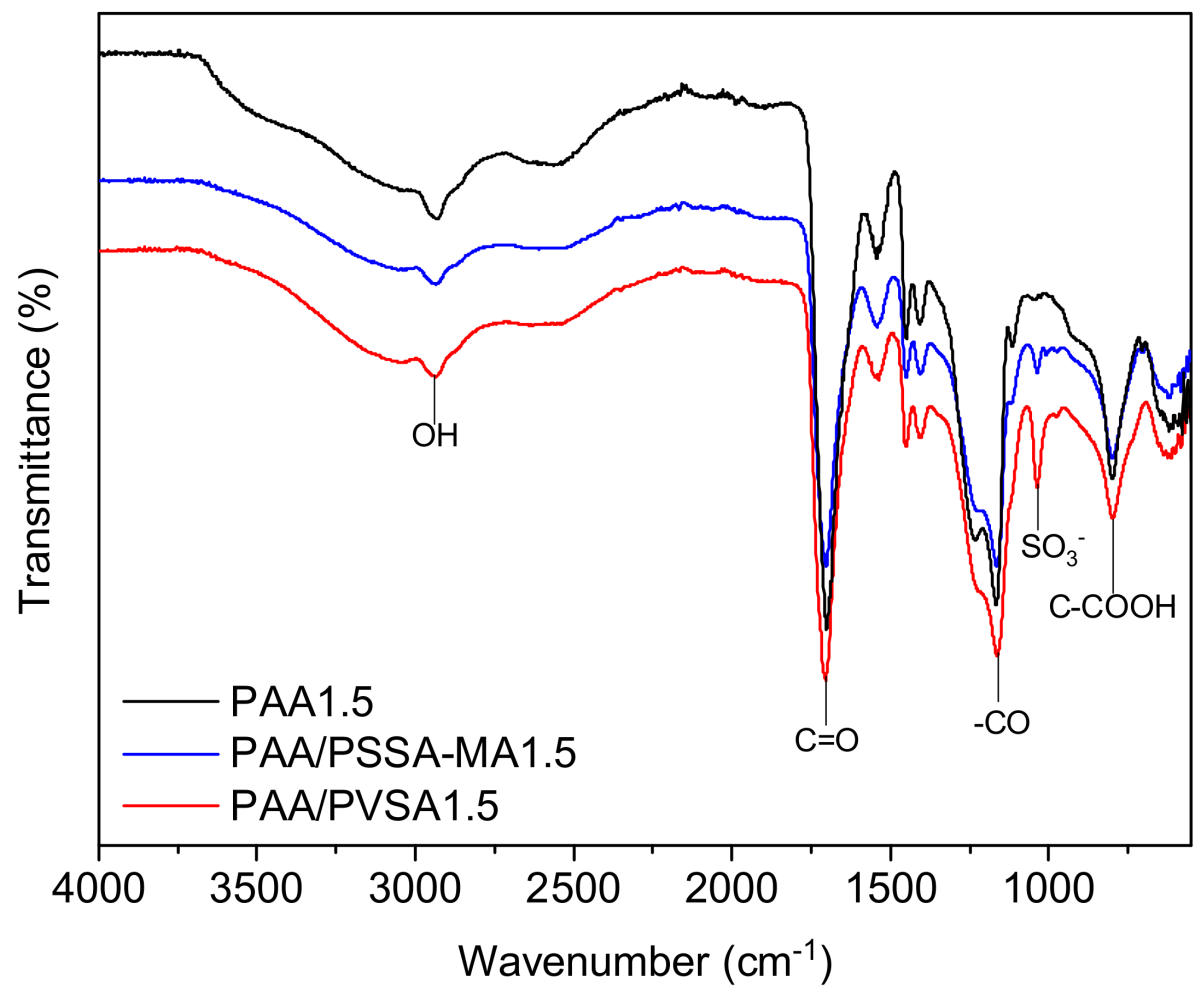
FTIR spectrum of PAA1.5, PAA/PVSA1.5, PAA/PSSA-MA1.5.

The chemical structure significantly affects the swelling ratio, mechanical properties and interactions with the aqueous environment, which are crucial factors for the energy recovery of the hydrogels. The chemical structure of the hydrogels was confirmed by FTIR as illustrated in Figure 5. The FTIR spectra of PAA showed an important strong sharp band at 1706 cm^−1^ which is due to vibration of carbonyl (–C=O) stretching of the –COOH groups of AA [59]. Broad absorption bands at 2932 cm^−1^ indicate –OH stretching vibration of carboxylic acid [56,60]. The strong vibration band between 1130 cm^−1^ and 1240 cm^−1^ is caused by (–CO) stretching and (–OH) bending of the –COOH groups [56,61]. The broad band at 796 cm^−1^ indicates C–COOH stretching [62]. The main variation in the FTIR spectra occurs at the absorption band at 1036 cm^−1^ which corresponds to S=O asymmetric stretching [56,60,63]. This confirms the presence of sulfonic groups in PAA/PVSA and PAA/PSSA. It was observed that the density of sulfonic groups in PAA/PVSA were higher than for PAA/PSSA-MA. This possibly indicates that part of PAA/PSSA-MA was released during the shrinking/swelling process [64].

### 3.4. Swelling Volume with Multi-Cycle Measurements

**Figure 6:**
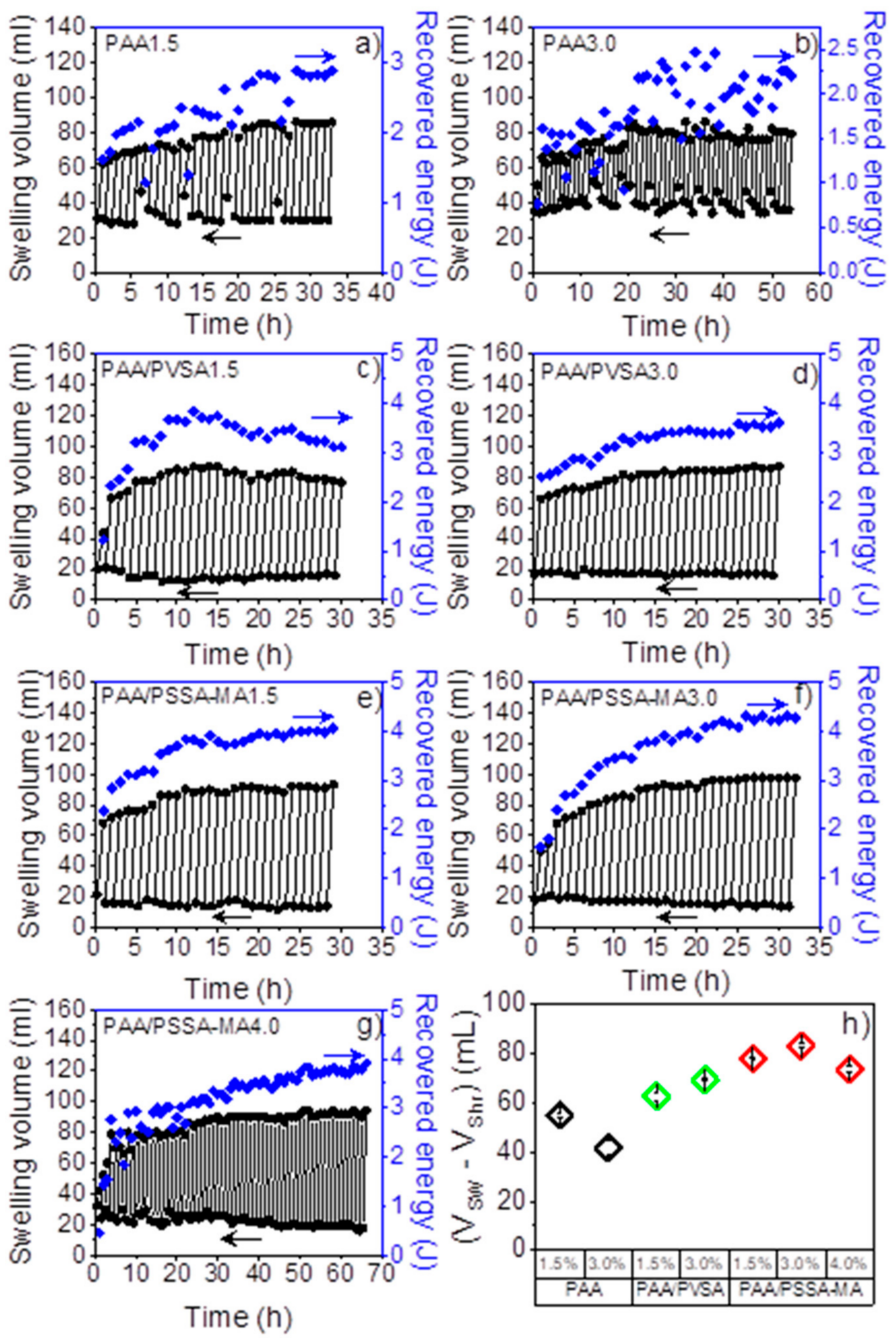
Repeated swelling cycles as a function of time: (**a**) PAA1.5; (**b**) PAA3.0; (**c**) PAA/PVSA1.5; (**d**) PAA/PVSA3.0; (**e**) PAA/PSSA-MA1.5; (**f**) PAA/PSSA-MA3.0; (**g**) PAA/PSSA-MA4.0; and (**h**) Average volume difference between swollen and shrunken volume of the 6 last cycles.

The swelling volume of the different hydrogels at varying crosslinker concentrations was investigated utilizing a 5 kg external load. One cycle of the energy recovery process includes: (i) shrinking when the hydrogels are exposed to a high salinity aqueous solution (35 g/L NaCl), and (ii) swelling when de-ionized water was pumped through the hydrogel. The swelling of the hydrogels lifts the external load, which produces potential energy. Several shrinking-swelling cycles were repeated as a function of time as shown in Figure 6a–g. Interestingly, the swelling volume of the hydrogels increases during the initial cycles, before it reaches a steady state after several cycles. Imran et al. [65] reported that a dense skin layer may form on the surface of the hydrogel when it shrinks, thereby inhibiting water loss from the inner portions. In addition, under the pressure of the heavy load, the hydrogel is broken into smaller pieces. Therefore, after several cycles, the swelling/shrinking process becomes faster. It is worthy to note that the swelling volume of the PAA-based hydrogels exceeds the initial swelling ratio (Figure 6a–g). This suggests that the composition of the gels may play an important role regarding the swelling of the hydrogels.

**Figure 7:**
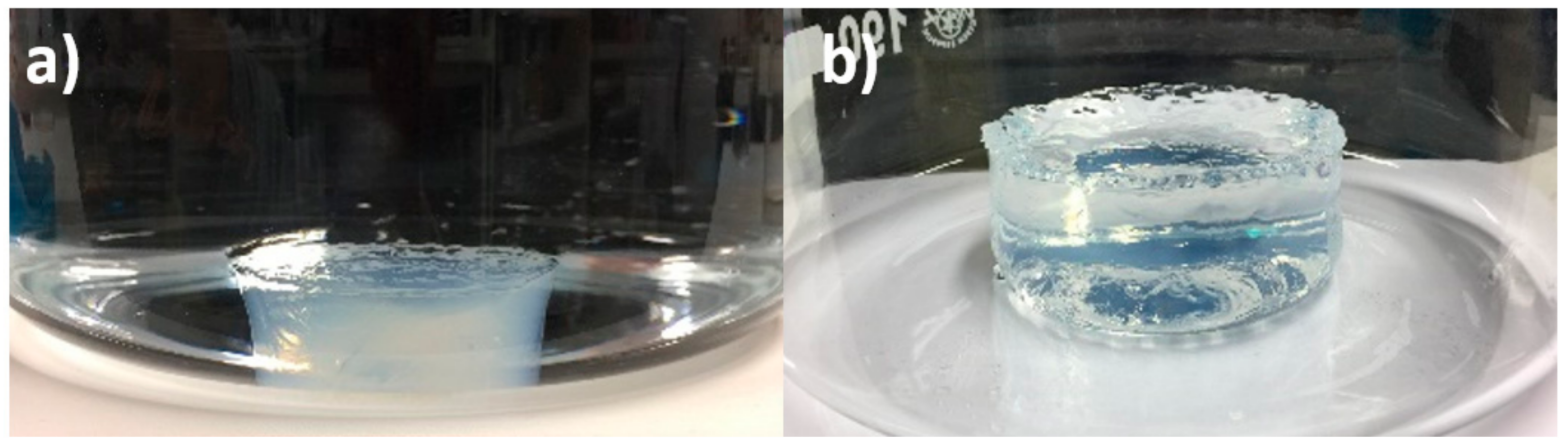
(**a**) Initial swelling of PAA1.5 hydrogel in de-ionized water (diameter: 64 mm, thickness: 22 mm); (**b**) swelling of PAA1.5 hydrogel for 8 days in 1.2 mol/L urea to reduce hydrogen boning (diameter: 86 mm, thickness: 33 mm).

The enhanced swelling of PAA-based hydrogels is probably due to the disassociation of hydrogen bonds by the stress [66] over repeated cycles. Hydrogen bonds act as additional crosslinking points in the gel network, thereby restricting the gel’s ability to swell. As the hydrogen bonds are gradually broken during repeated cycling, the maximum swelling volume increases. In order to confirm that the gels are affected by hydrogen bonds, the PAA1.5 gel was swelled in 1.2 M urea. Since urea is a hydrogen bond breaker, this will illustrate the effect of reducing hydrogen bonds in the gels. The hydrogel that is swollen in 1.2 M urea (Figure 7b) is much less turbid than the same gel swollen in de-ionized water (Figure 7a). In addition, the hydrogel swells to a larger size in urea (86 mm × 33 mm) than in de-ionized water (64 mm × 22 mm). The reduced turbidity is a clear sign that the gels have fewer associated microdomains between the polymer chains, i.e., less crosslinks to restrict the gels from swelling. These findings support the conjecture that the enhanced swelling of the gels with increased number of swelling cycles is caused by a reduction of hydrogen bonded physical crosslinks.

Figure 6h shows the average swelling volume calculated from the difference between the minimum shrinking volume and maximum swelling volume of the 6 last cycles. The PAA hydrogels exhibit a slow shrinking process (25–30 min) compared to PAA/PVSA and PAA/PSSA-MA (10–15 min) (data not shown). The low swelling ratio of the PAA hydrogel (Figure 2) can be attributed to high amounts of crosslinks and hydrogen bonds. The presence of sulfonic acid groups in PAA/PSSA-MA (Figure 6e–g) and PAA/PVSA (Figure 6c,d) was found to speed up the shrinking process as well as to increase the swelling volume, due to completely dissociated sulfonic acid groups. The charged groups facilitate a rapid response of both the salting-out effect and of the electrostatic inter-chain repulsion.

**Figure 8:**
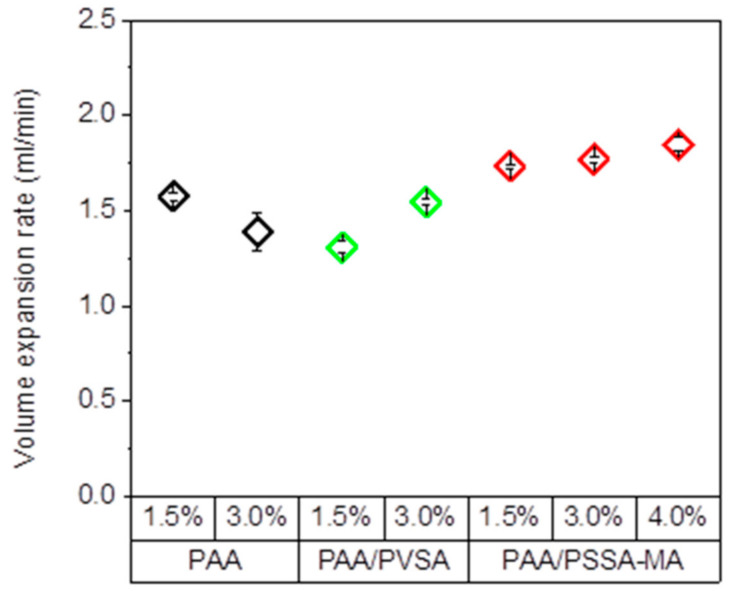
Lifting rate of a 5 kg weight by the different hydrogels.

The PAA/PSSA-MA hydrogels exhibit higher (Figure 2) and faster (Figure 8) shrinking and swelling than the PAA/PVSA hydrogels. The flexibility of the linear polyelectrolyte PSSA-MA interpenetrating in the PAA network results in increased electrostatic interactions. The sulfonic groups on the immobilized copolymer PAA/PVSA backbone decrease the flexible electrostatic interactions of the –SO_3_^−^ groups, resulting in a lower swelling volume of PAA/PVSA (Figure 6h). It is worth noting that PAA/PSSA-MA4.0 (Figure 6g) exhibited a constantly increasing swelling volume when the cycles were repeated up to 66 cycles. Figure 6h shows that swelling volume of PAA/PSSA-MA exhibit a maximum when the crosslinker density is increased, since high amounts of crosslinks restricts the swelling ability of the hydrogel.

### 3.5. Volume Expansion Rate

Swelling volume expansion rate is an important factor for the energy that can be extracted from the system. Functional groups in the polymer network and the crosslinking density of the hydrogels causes variations in swelling rate. The swelling volume expansion rate is defined as the expansion volume divided by the time between the minimum and maximum volume of the hydrogels after 30 repeated cycles. As expected, the presence of the highly negatively charged SO_3_^−^ in the PAA /PSSA-MA network increase the electrostatic interactions with the COO^−^ groups, and leads to an increased expansion rate of the hydrogel compared to PAA (Figure 8). Additionally, the PAA/PSSA-MA hydrogels exhibit a faster expansion rate than the PAA/PVSA hydrogels. It is possible that the large PSSA-MA molecules penetrating in the PAA network give rise to a less compact structure than for the PAA/PVSA hydrogel. Additionally, the free segment motion of the semi-interpenetrated polymer may trigger more free spaces within the polymeric system leading to an enhancement of the migration of water [55,67].

For PAA, increasing the crosslinker concentration results in a slower expansion rate of the hydrogel (Figure 8). However, interestingly, the expansion rate of PAA/PVSA becomes higher as the crosslinking concentration is raised. The reason for this is currently unclear.

### 3.6. Recovered Energy and Energy Efficiency

**Figure 9:**
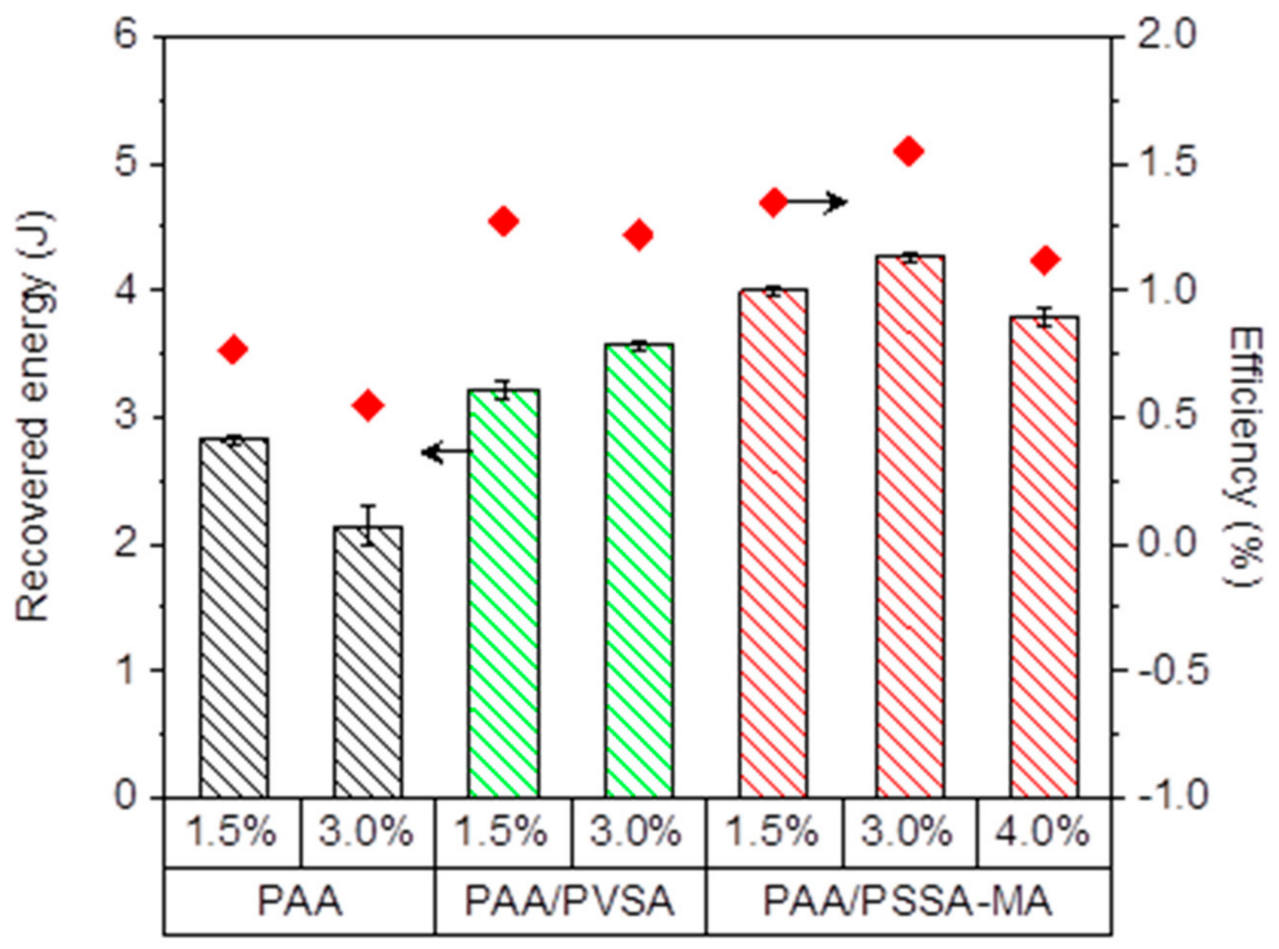
Recovered energy and energy efficiency as a function of crosslinker concentration of the hydrogels.

Figure 9 shows the recovered energy and energy efficiency as a function of crosslinker concentration of the hydrogels. The highest recovered energy was obtained by the PAA/PSSA-MA hydrogel with up to 4 J in one cycle.

Increasing the crosslinker concentration significantly affected the energy recovery capability of the hydrogels. Figure 9 illustrates that higher crosslinker concentrations increases the recovered energy for PAA/PVSA and PAA/PSS-MA when the crosslinker concentration is raised from 1.5% to 3.0%. However, a further increase of the crosslinker concentration results in a more compact network structure where swelling is restricted by an excess of crosslinking points. Consequently, the recovered energy goes through a maximum when the crosslinking density is increased, similar to what has been observed previously for other hydrogels [30,31,32]. At low crosslinking concentrations, additional crosslinks render the network stronger, enabling it to lift the heavy weight higher (Figure 6h), and thereby recover more energy. When the crosslinker concentration becomes too high, the space between crosslinks becomes too short, which restricts the swelling of the hydrogels (Figure 2).

The recovered energy is a result of the swelling ability of the hydrogel under external load, which is dependent on electrostatic interactions and the morphological architecture of the charged groups in the hydrogel network. PAA/PSSA-MA recovered more energy than PAA/PVSA. This suggest that semi-IPN hydrogels which facilitates motion of some of the charged groups, may promote energy recovery.

**Figure 10:**
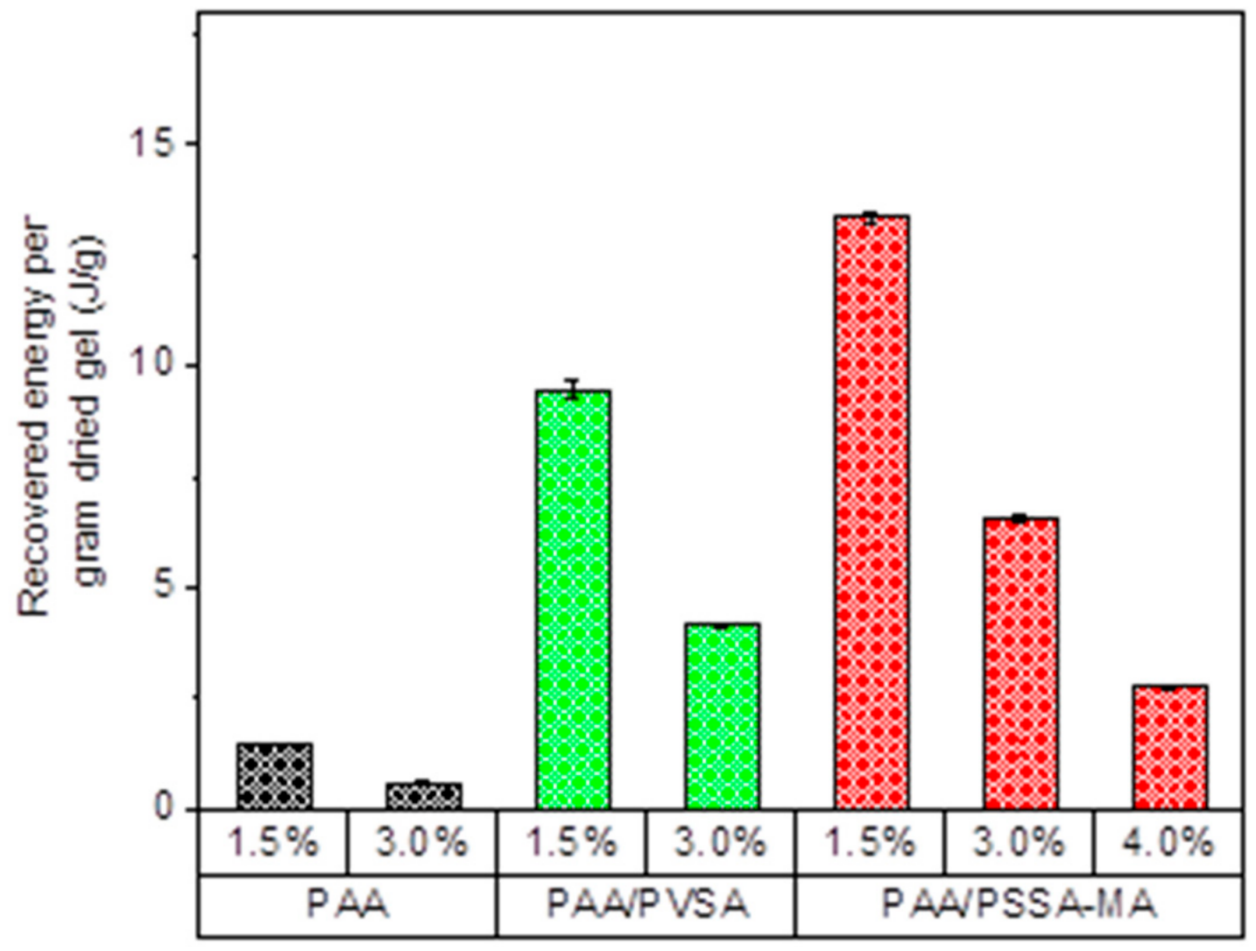
The maximum recovered energy per gram of dried hydrogel for the different hydrogels.

Figure 10 depicts the equilibrium recovered energy per gram of dried hydrogel as function of crosslinker density of the hydrogels. The recovered energy per gram of dried hydrogel is reduced as the crosslinker concentration is raised for all three types of PAA-based hydrogels. The amount of energy produced per gram of dried hydrogel is up to 13.3 J/g (3.7 mW/g) for PAA/PSSA-MA1.5.

Comparing the produced energy of these hydrogels with previous studies (Table 2), it is clear that the PAA/PSVA and PAA/PSSA-MA produces more energy than most of the previously studied systems (except for polysulfobetaine, which exhibits a very high energy production). However, the produced energy is not only dependent on the properties of the hydrogels themselves, but also on all the parameters of the measuring system, such as applied external load, pumping rates of the high and low salinity solutions, and syringe dimensions, as well as the amount of hydrogel used and the size of the hydrogel particles [29,30,31,32]. It is therefore difficult to directly compare the values from different studies, especially when they originate from several research groups. This illustrates the importance of comparing several hydrogel systems within the same study, in order to clearly separate the effect of the initial properties of the hydrogels from variations due to the experimental setup.

## 4. Conclusions

Energy from salinity gradients can be generated from expansion and contraction of hydrogels. In this study, PAA, PAA/PVSA, and PAA/PSSA-MA hydrogels were used to recover the mixing energy from saline and water. When the polymer network structure becomes very tight at high crosslinking densities, the swelling capacity is reduced. The presence of strongly charged functional groups enhance the swelling ratio. The hydrogels could maintain the recovering process for 30 repeated cycles, and for some of the hydrogels, the recovered energy increased after repeated cycles. The swelling rate of the copolymer network and semi-interpenetrating network is higher than that of PAA hydrogels. The energy efficiency reaches 1.6%. The maximum amount of energy produced per gram of polymer was 13.3 J/g, which is a significant improvement compared to our previous study (3.4 J/g) [31]. These results are a promising step towards future applications for generating green energy. However, before assessing its practical use, there is a need for scaling up the system and building an osmotic engine that can extract the produced energy.

## Figures and Tables

**Table 1:** The composition of AA, VSA and PSSA-MA in the PAA, PAA/PVSA, PAA/PSSA-MA hydrogels. The numbers in the sample denotations indicate the molar crosslinker concentrations.

Sample Denotation	AA (g)	VSA (mL)	PSSA-MA (g)	MBA (g)	APS (g)	TEMED (mL)	Gelation Time (min)
PAA1.5	2.5	-	-	0.08	0.04	0.24	120
PAA3.0	2.5	-	-	0.16	0.04	0.24	40
PAA/PVSA1.5	2.5	4	-	0.10	0.04	0.24	120
PAA/PVSA3.0	2.5	4	-	0.20	0.04	0.24	60
PAA/PSSA-MA1.5	2.5	-	1	0.08	0.04	0.24	120
PAA/PSSA-MA3.0	2.5	-	1	0.16	0.04	0.24	60
PAA/PSSA-MA4.0	2.5	-	1	0.21	0.04	0.24	40

**Table 2:** Comparison of the energy recovered from different hydrogels.

Hydrogel Type	Recovered Energy per Gram dry Hydrogel (J/g)	Reference
PAA	0.34	[29]
PAA	0.6	current study
PAA	0.83	[30]
poly(allylamine hydrochloride)	3.4	[31]
PAA/PSVA	9.5	current study
PAA/PSSA-MA	13.3	current study
polysulfobetaine	102	[32]

## Data Availability

The measured data cannot be shared at the current time as the data also forms part of an ongoing study.
